# Effect of flow-optimized pressure control ventilation-volume guaranteed (PCV-VG) on postoperative pulmonary complications: a consort study

**DOI:** 10.1186/s13019-024-02881-x

**Published:** 2024-07-08

**Authors:** Ting Ting Sun, Ke Xin Chen, Yong Tao, Gong Wei Zhang, Li Zeng, Min Lin, Jing Huang, Yue Hu

**Affiliations:** Department of Anesthesia Operation, The First People’s Hospital of Shuangliu District (West China Airport Hospital of Sichuan University), No.120, Chengbei Street, Dongsheng Street, Shuangliu District, Chengdu, 610200 China

**Keywords:** Inspiratory pressure rise time, One-lung ventilation, PCV-VG ventilation, Thoracic surgery, Postoperative pulmonary complications

## Abstract

**Background:**

Postoperative pulmonary complications (PPCs) after one-lung ventilation (OLV) significantly impact patient prognosis and quality of life.

**Objective:**

To study the impact of an optimal inspiratory flow rate on PPCs in thoracic surgery patients.

**Methods:**

One hundred eight elective thoracic surgery patients were randomly assigned to 2 groups in this consort study (control group: *n* = 53 with a fixed inspiratory expiratory ratio of 1:2; and experimental group [flow rate optimization group]: *n* = 55). Measurements of Ppeak, Pplat, PETCO_2_, lung dynamic compliance (Cdyn), respiratory rate, and oxygen concentration were obtained at the following specific time points: immediately after intubation (T0); immediately after starting OLV (T1); 30 min after OLV (T2); and 10 min after 2-lung ventilation (T4). The PaO_2_:FiO_2_ ratio was measured using blood gas analysis 30 min after initiating one-lung breathing (T2) and immediately when OLV ended (T3). The lung ultrasound score (LUS) was assessed following anesthesia and resuscitation (T5). The occurrence of atelectasis was documented immediately after the surgery. PPCs occurrences were noted 3 days after surgery.

**Results:**

The treatment group had a significantly lower total prevalence of PPCs compared to the control group (3.64% vs. 16.98%; *P* = 0.022). There were no notable variations in peak airway pressure, airway plateau pressure, dynamic lung compliance, PETCO_2_, respiratory rate, and oxygen concentration between the two groups during intubation (T0). Dynamic lung compliance and the oxygenation index were significantly increased at T1, T2, and T4 (*P* < 0.05), whereas the CRP level and number of inflammatory cells decreased dramatically (*P* < 0.05).

**Conclusion:**

Optimizing inspiratory flow rate and utilizing pressure control ventilation -volume guaranteed (PCV-VG) mode can decrease PPCs and enhance lung dynamic compliance in OLV patients.

## Introduction

Postoperative pulmonary complications (PPCs) significantly impact patient prognosis and quality of life [1–3]. General anesthesia contributes to atelectasis, leading to intraoperative hypoxemia, which in turn results in postoperative pulmonary adverse events and is linked to PPCs. The choice of ventilation mode does not impact the likelihood of PPCs in patients undergoing pulmonary resection with one-lung ventilation (OLV) [2]. PPCs occur in approximately 22–43% of patients undergoing OLV [1–3]. During OLV, patients often have intraoperative hypoxemia and PPCs because of pulmonary shunting and atelectasis [4, 5]. PPCs occur in up to 59% of thoracic surgery patients [4]. Thoracic surgical injury and one-lung breathing traction trigger a potent immunologic response in the lung endothelium and alveoli. The immunologic response causes the release of inflammatory substances that leads to an excessive buildup of neutrophils, which increases pulmonary vascular permeability and ultimately causes lung injury [6].

The primary goals of setting and adjusting ventilator settings during mechanical ventilation are to provide proper ventilation and oxygenation, prevent issues connected to the ventilator, and enhance ventilation efficiency. Prior research has shown that utilizing a lung-protective ventilation technique decreases the occurrence of PPCs, corrects an ventilation: perfusion ratio imbalance, enhances oxygen levels while on mechanical ventilation, and enhances long-term outcomes. Anesthesiologists typically focus on tidal volume, airway pressure, and corresponding curves during mechanical ventilation. Utilizing decreased tidal volume, positive end-expiratory pressure, and lung recruitment is advisable during the perioperative phase [7]. However, this approach may not be entirely appropriate for patients undergoing pneumonectomy. Using a tidal volume of 6 ml/kg instead of 10 ml/kg for ventilation provides lung protection [8, 9].

Nevertheless, employing low tidal volume without sufficient positive end-expiratory pressure (PEEP) could increase the risk of atelectasis and result in higher PPCs [10]. Customized PEEP enhances oxygen levels, end-tidal lung volume (EELV), and respiratory function when ventilating patients during surgery. Nevertheless, these benefits may diminish shortly after removing the endotracheal tube [11–13]. Using driving pressure-guided ventilation during OLV decreases PPCs compared to traditional lung protective ventilation [14]. Dynamic pressure-guided PEEP is user-friendly during surgery but is susceptible to several factors, including changes in body position. Whether a lung-protective ventilation approach or driving pressure-guided pulmonary ventilation is used, the effect on pulmonary problems is transient and can be quickly nullified.

Although inspiratory-expiratory flow curves offer valuable information, inspiratory-expiratory flow curves are often overlooked. Flow curves during mechanical ventilation provide valuable data on respiratory mechanics, patient resistance, and patient-ventilator interactions, which are essential for adjusting ventilator settings. The results in a study show that by using a flow optimization ventilation strategy, pressure control ventilation (PCV) with a goal of “zero flow at the end of inspiration” significantly improved intraoperative respiratory mechanics [15]. The underlying principle is as follows: a preset pressure level is reached in PCV with a high initial inspiratory flow at the beginning of the inspiratory process; and this pressure level decreases exponentially as the inspiratory time increases until the end of the inspiratory process. The end-inspiratory flow rate is adjusted to “0” by adjusting the inhalation: exhalation ratio to more accurately adjust the ventilation strategy during PCV ventilation. “Zero flow rate at the end of inhalation” means that the inspiratory time is appropriate and slightly longer. Under this ventilation strategy, atelectasis caused by hypopnea can be effectively reduced and alveolar hyperinflation caused by hyperventilation can be avoided [15].

### Objectives

How can we adjust the ventilation parameters based on the flow curve to minimize PPCs without changing the ventilation mode? We designed the current study to investigate this issue. Pressure control ventilation-volume guaranteed (PCV-VG) was used by adjusting the inspiratory rise time so that the air flows through the airway at a uniform speed during inhalation to reduce the end-inspiratory flow rate and make the gas diffusion more uniform. We determined if this method reduced air damage to the airway, improved the dynamic compliance of the lung, and reduced lung complications after single lung ventilation.

## Methods

### Study design

This consort study was an investigator-initiated, single-center, randomized controlled, patient- and evaluator-blinded trial. The study was conducted in compliance with the Declaration of Helsinki (updated in 2013) and authorized by the Ethics Committee of The First People’s Hospital of Shuangliu District, Chengdu City (No. 2022-4-01 with approval on 25 October 2022). Clinical trial registration on https://www.chictr.org.cn/ (No. ChiCTR2300071367) was completed on 12 May 2023. Each participant provided written informed consent.

### Participants

One hundred eight patients undergoing thoracic surgery with OLV under general anesthesia were enrolled between May and December 2023. Based on a random number table, all eligible patients were randomly allocated into a control group with a fixed inspiratory: expiratory ratio of 1:2 and an experimental group with flow rate optimization according to the random number table. The criteria for inclusion were as follows: 1) adult patients undergoing elective lobectomy with an estimated OLV time of at least 30 min; 2) *≥* 19 years of age and ASA I-III; 3) voluntary participation in the study with signing of the informed consent form; 4) no history of mental illness (depression and schizophrenia), drug abuse, and alcohol addiction; and 5) not enrolled in any other studies. The exclusion criteria were as follows: (1) heart failure; (2) pulmonary bullae and emphysema; (3) pregnant or lactating women; (4) receiving oxygen therapy; (5) presence of other significant conditions that prevent surgery; and (6) single lung ventilation time < 30 min.

### Anesthetic methods (interventions)

Pulmonary ultrasonography was utilized to assess the patient’s pulmonary status before surgery. The blood pressure, heart rate, oxygen saturation, an electrocardiogram, and the bispectral index (BIS) were recorded after surgery. The radial artery was cannulated after administration of a local anesthetic and invasive arterial blood pressure was continuously monitored. Both groups received the same drugs for anesthesia induction and maintenance (sufentanil [0.5 µg/kg], propofol [2 mg/kg], and cis-rocuronium [0.15 mg/kg] intravenously). Anesthesia maintenance included propofol at a rate of 6–8 mg/(kg·h), desflurane inhalation at 4–5%, remifentanil at 0.15–0.25 µg/(kg·min), and intermittent injections of cis-atracurium to ensure adequate muscle relaxation. BIS readings fluctuated between 40 and 60 during maintenance. Following anesthetic induction, all patients had double-lumen endotracheal intubation (male, 35 F and female, 32 F; WELL LEAD MEDICAL CO., LTD., Guangzhou, China). The catheter was repositioned using fiberoptic bronchoscopy and attached to the anesthetic machine (Mindray A8C/A8 pro; Shen zhen, China) set to ventilate in the PCV-VG mode. The mechanical ventilation settings were as follows: control group, two-lung ventilation with tidal volume of 8 ml/kg, OLV with tidal volume of 6 ml/kg, fresh air flow of 2 L/min, end-expiratory positive pressure (PEEP) set at 5 cmH_2_O, end-expiratory carbon dioxide partial pressure (PETCO_2_) maintained between 30 and 55 mmHg, pressure rise time adjusted to 0.2 s (ventilator default), and a fixed inspiratory expiratory ratio of 1:2; and experimental group, tidal volume for two-lung ventilation, 8 ml/kg for total volume, 6 ml/kg for OLV, 5cmH_2_O for PEEP, and PETCO_2_ maintained between 30 and 55 mmHg. The pressure rise and inspiratory time were adjusted based on pressure and flow waveforms. During PCV-VG, the airway pressure waveform has a plateau period, which is presented as “battlement-like.” However, if the pressure rise time at the initial stage of aspiration is very short, a large amount of gas will be transported in a short period of time, resulting in turbulent air flow at the initial stage of aspiration. The airway pressure waveform appears as a creped-like bulge at the early stage of the plateau period and the curve is presented as “jagged” or “irregular.” At this time, the pressure waveform can be presented as “battlements” by adjusting the suction pressure rise time. The flow waveform during PCV is an exponential decreasing wave, which is characterized based on the flow delivered by the ventilator immediately peaking at the beginning of the inspiratory time, then decreasing exponentially to “0” (the end of the inspiratory time). The inspiratory flow gradually decreases to “0” or close to “0” by adjusting the inspiratory time or the inhalation-to-breath ratio. A plat < 30 cmH_2_O was established as the threshold to prevent barotrauma. Goal-directed fluid treatment (GDFT) was used for fluid resuscitation. The tidal volume was set between 8 and 10 ml/kg during two-lung ventilation. The pulse pressure variation rate was maintained at *≥* 13% or the stroke volume variation (SVV) was at *≥* 12%, the patient should be rehydration. When using a tidal volume of 6 ml/kg, the pulse pressure variation rate was at *≥* 6% or the SVV at *≥* 8%, the patient should be rehydration [16]. The blood pressure was kept within ± 20% of the preoperative resting blood pressure fluctuation, while the heart rate ranged from 60 to 100 beats per minute during the procedure. Cardiovascular active medications were administered in response to hypotension or bradycardia during the procedure. Desflurane and remifentanil were discontinued after surgery and the airflow was set to 8 L/min. The endotracheal tube was removed when the patient regained spontaneous breathing, consciousness, and protective reflexes.

### Data collection: primary and secondary outcomes

Baseline data collection included gender, age, height, weight, and ASA classification.

The primary outcomes measurements were incidence of atelectasis immediately after surgery. Pulmonary sonography was performed and the lung ultrasound score (LUS) was used to evaluate the occurrence of atelectasis.

The secondary outcome measurements were incidence of pulmonary complications within 3 days post-operatively was documented [1], including respiratory tract infections, atelectasis, and pleural effusions. The respiratory mechanics index included airway peak pressure (Ppeak), airway plateau pressure (Pplat), and PEEP. The ventilation index included the PETCO_2_, dynamic lung compliance, and the oxygenation index (PaO_2_/FiO_2_). A respiratory infection was defined as a patient who received antibiotics for a suspected respiratory infection and met one or more of the following criteria: new or altered sputum; new or altered lung opacity; fever (body temperature ≥ 38 °C); and increased white blood cell (WBC) count (＞12 × 10^9^/L). Atelectasis was defined as lung opacity, movement of the mediastinum, hilum, or diaphragm towards the affected area, and compensatory hyperinflation of the adjacent non-atelectasis lung, as confirmed by X-ray. Pleural effusion was defined as evidence of a blunted costophrenic angle on a chest radiograph, loss of a sharp contour of the ipsilateral diaphragm in an upright position, and displacement of adjacent anatomic structures requiring clinical treatment. Ppeak, Pplat, PETCO2, pulmonary dynamic compliance (Cdyn), respiratory rate, and oxygen uptake concentration were measured at the following specific time points: immediately after intubation (T0); immediately after starting OLV (T1); 30 min after OLV (T2); and 10 min after two-lung ventilation (T4). Blood gas analysis was performed 30 min after OLV (T2) and immediately when OLV ended (T3) to measure the PaO_2_/FiO_2_. LUS was assessed upon arrival at the anesthesia resuscitation room (T5). After removing the nasal cannula for oxygen inhalation within 3 days postoperatively, a diagnosis of hypoxemia was made if the oxygen saturation was < 90% during inhalation. The nasal cannula for oxygen inhalation was replaced for respiratory support or a non-invasive ventilator was used in patients with hypoxemia. On the initial postoperative day, number of inflammatory cells and CRP level were documented. An ultrasound was performed at the T5 level to evaluate the occurrence of atelectasis in the contralateral lung [17, 18]. Atelectasis was diagnosed if the LUS scores was > 2.

### Statistical methods

PASS2021 software was used for sample estimation. The incidence of postoperative pulmonary complications in thoracic surgery patients has been reported to be as high as 59% [4]. We hypothesized that the optimal flow rate ventilation parameter settings would further reduce the incidence of pulmonary complications after single lung ventilation to 34%; specifically, the incidence of primary atelectasis would be reduced by 25% with an α = 0.05, test efficacy (1-β) of 0.80, and lost to follow-up rate of 5%. The sample size of each group was calculated to be 51.

All patients meeting the inclusion criteria were randomly divided into test and control groups according to a random number table.

This study used a double-blind method. Therefore, the attending anesthesiologist was aware of the designated group, but the subject and evaluator did not know which mechanical ventilation mode was used.

The data analysis for this study was conducted using IBM SPSS Statistics 26.0 software (IBM, Armonk, USA). Statistical significance was determined at a *P* < 0.05. Normality tests were performed on all continuous variables. Data following a normal distribution are presented as the mean ± standard deviation, while data not following a normal distribution are presented as the median. Continuous variables were assessed using the Student’s t-test to compare groups. The Pearson χ2 test was used for categorical data with rates and percentages, whereas the Kruskal-Wallis test was used for ordinal categorical variables. For continuous variables with a non-normal distribution, the Mann-Whitney U test was selected.

## Results

### Baseline data

**Fig. 1:**
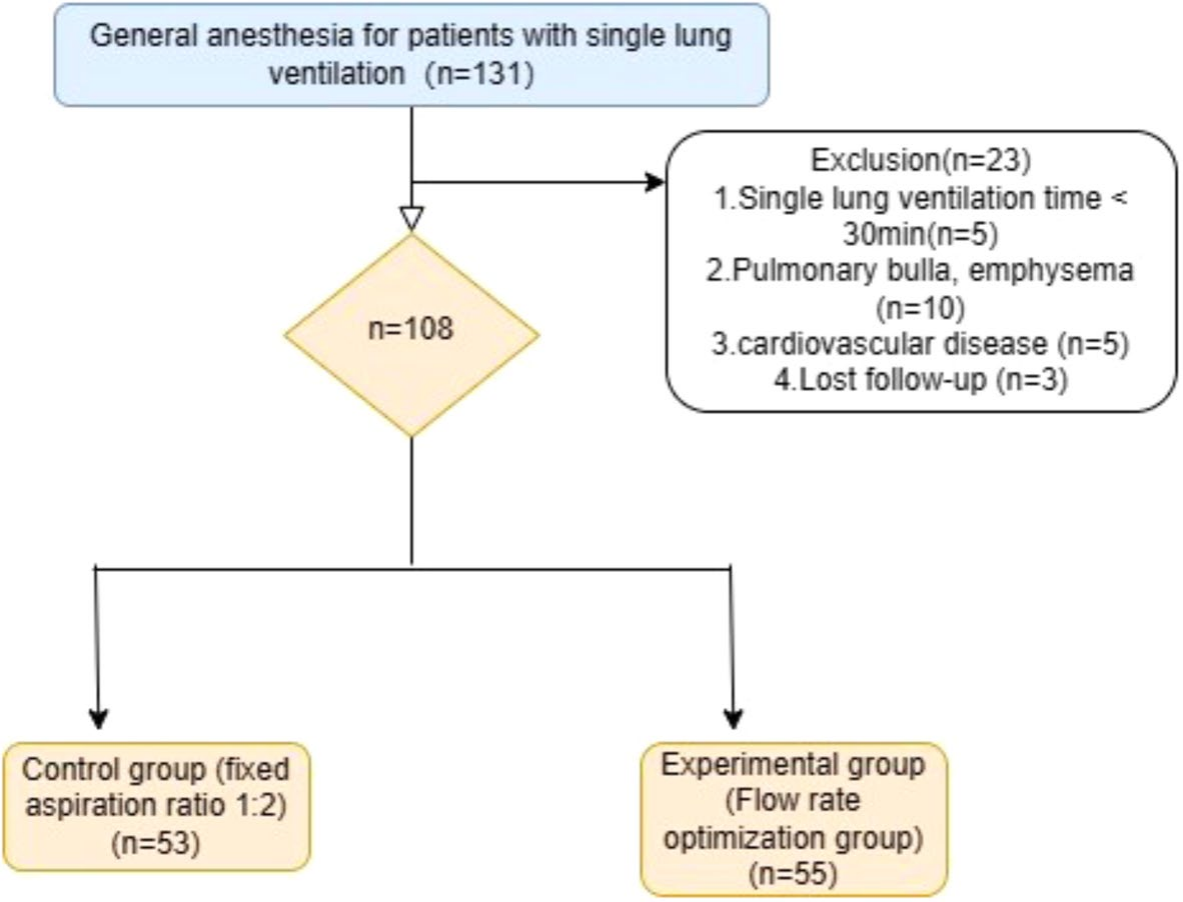
The study enrollment flow chart

Figure 1 displays the enrollment flow chart for this study. A total of 108 patients were randomly placed into control (*n* = 53) and experimental groups (*n* = 55). There were no notable differences in the following data between groups: age; gender; body mass index; ASA classification; operative time; anesthesia time; intraoperative blood loss; and perioperative transfusion rate (*P* > 0.05). The total intraoperative infusion volume in the experimental group (flow rate optimization group) was significantly lower than the control group (*P* = 0.028; Table 1).

**Table 1:** Fundamental situation analysis of each group of patients. values are the median (IQR [range]) or mean (SD)

	Control group (*n* = 53)	Experimental group (*n* = 55)	t/x^2^ value		*P* value
Age (years)	50.62 ± 6.75	53 ± 10.16	0.550		0.446
Gender (male: female)	26:27	29:26	3.236		0.087
Body mass index (kg/m2)	22.91 ± 2.64	23.83 ± 3.13	0.645		0.978
ASA classification					
II	50	51	2.271		0.331
III	3	2			
Anesthesia time (min)	148.12 ± 23.78	121.87 ± 11.79	0.989		0.218
Operative time (min)	102.25 ± 21.44	77.75 ± 12.59	0.985		0.346
Amount of intraoperative bleeding (mL)	21.25 ± 11.25	16.25 ± 4.97	0.406		0.278
Intraoperative fluid replacement (mL)	997.5 ± 120.29	812.5 ± 62.5	1.365		0.028*

Data are represented as n (%) or mean ± SD. ASA, American Society of Anesthesiologists; SD, standard deviation; *P<0.05

### Occurrence of atelectasis immediately after surgery

Patients were admitted to the anesthesia recovery room postoperatively and the occurrence of atelectasis was evaluated by LUS. The incidence of atelectasis immediately after surgery in the experimental group decreased to 1.82% compared to 5.67% in the control group (*P* = 0.291; Table 2).

**Table 2:** Comparison of PPC incidence between the two groups

Group	Control group (*n* = 53), n (%)	Experimental group (*n* = 55), n (%)	x^2^ value		*P* value
Respiratory infection	4(7.55)	1(1.82)	2.006		0.157
Atelectasis	4(7.55)	1(1.82)	2.006		0.157
Pleural effusion	1(3.77)	0(0)	2.115		0.146
Respiratory failure	0(0)	0(0)	-		-
Overall incidence of PPCs	9(16.98)	2(3.64)	5.254		0.022*
Hypoxemia within 72 h of surgery	3(5.67)	1(1.82)	1.117		0.291
Atelectasis immediately after surgery (LUS>2)	3(5.67)	1(1.82)	1.117		0.291

PPCs, postoperative pulmonary complications; *P<0.05

### Occurrence of PPCs

The incidence of PPCs in patients with single lung ventilation after thoracic surgery within 3 days after surgery was 3.64% and 16.98% in the experimental and control groups (*P* = 0.022; Table 2).

### Comparison of respiratory mechanics-related indices between the two groups at different time intervals

At baseline (T0), there were no statistically significant differences between the two groups in Ppeak, Pplat, Cdyn, PETCO_2_, respiratory rate, and oxygen concentration (*P* > 0.05). The experimental group (flow rate optimization group) showed a substantial increase in Cdyn at T1, T2, and T4 compared to the control group (*P* < 0.05). Additionally, the PaO_2_/FiO_2_ at T3 was significantly higher in the experimental group (*P* < 0.05; Table 3).

**Table 3:** Comparison of respiratory mechanics-related indices between the two groups at different time points

Group	Control group (*n* = 53)	Experimental group (*n* = 55)	T value	*P* value
Airway peak pressure (cmH_2_O)				
T0	12.12 ± 1.35	13.75 ± 2.54	1.592	0.134
T1	19.01 ± 2.87	18.12 ± 4.12	0.492	0.630
T2	18.12 ± 3.22	18.75 ± 4.20	0.344	0.744
T4	13.50 ± 3.58	12.62 ± 2.97	0.531	0.604
Airway plateau pressure (cmH_2_O)				
T0	11.75 ± 1.16	13.50 ± 2.72	1.670	0.117
T1	18.12 ± 3.09	18.00 ± 4.03	0.070	0.964
T2	18.33 ± 2.08	18.64 ± 3.01	0.453	0.675
T4	12.37 ± 2.61	12.62 ± 2.97	0.179	0.861
PET CO_2_ (mmHg)				
T0	38.25 ± 5.17	38.87 ± 5.89	0.225	0.825
T1	36.00 ± 3.07	34.75 ± 6.98	0.447	0.662
T2	39.12 ± 3.28	37.87 ± 2.08	0.322	0.753
T4	38.50 ± 1.41	35.37 ± 2.53	1.077	0.300
Pulmonary dynamic compliance (mL/cmH_2_O)				
T0	52.25 ± 7.99	54.75 ± 5.09	0.246	0.796
T1	32.37 ± 3.04	43.00 ± 3.02	2.478	0.027*
T2	35.62 ± 2.86	45.62 ± 3.05	2.386	0.032*
T4	44.01 ± 3.66	63.87 ± 8.31	2.187	0.046*
Respiratory rate				
T0	12.12 ± 1.24	12.50 ± 1.06	0.646	0.529
T1	13.25 ± 1.17	13.12 ± 1.55	0.182	0.858
T2	13.12 ± 0.99	13.50 ± 2.39	0.410	0.688
T4	12.75 ± 0.46	12.25 ± 1.28	1.038	0.317
Inhaled oxygen concentration (%)				
T0	68.25 ± 1.24	78.75 ± 4.68	1.372	0.192
T1	64.50 ± 4.07	68.87 ± 2.29	0.936	0.365
T2	70.37 ± 4.82	71.01 ± 4.07	0.095	0.926
T4	75.01 ± 4.93	71.87 ± 4.41	0.472	0.644
Oxygenation index				
T2	98.96 ± 29.12	95.37 ± 32.31	0.233	0.819
T3	173.73 ± 35.59	278.67 ± 30.34	2.244	0.042*

Data are represented as the mean ± SD. PETCO_2_, partial pressure of end-tidal carbon dioxide; SD, standard deviation; *P<0.05

### Comparing the WBC counts and C-reactive protein (CRP) levels in the two groups at different intervals

The experimental group (flow rate optimization group) had significantly lower WBC counts and CRP levels on postoperative day 1 compared to the control group (*P* < 0.05; Table 4).

**Table 4:** Comparison of WBC (x 10^9^) and CRP levels at different time points between the the two groups

Group	Control group (*n* = 53)	Experimental group (*n* = 55)	χ2 value	*P* value
WBC (x 10^9^)				
Preoperatively	5.28 ± 1.51	4.95 ± 1.49	0.443	0.665
1 day Postoperatively	12.14 ± 1.25	7.01 ± 0.53	3.777	0.002*
CRP (mg/L)				
Preoperatively	3.68 ± 1.79	6.13 ± 4.10	0.545	0.594
1 day Postoperatively	44.25 ± 6.83	27.52 ± 2.49	2.301	0.047*

Data are represented as the mean ± SD. WBC, white blood cell count; CRP, C-reactive protein; SD, standard deviation; *P<0.05

## Discussion

In the current study the treatment group had a significantly lower total prevalence of PPCs compared to the control group. No significant difference was detected in the occurrence of atelectasis immediately after surgery between the two groups. The Cdyn and PaO_2_/FiO_2_ were considerably increased at T1, T2, and T4, and the CRP level and inflammatory cells decreased dramatically. The current study showed that it is possible to achieve enhanced respiratory mechanics indices and enhanced lung compliance by modifying the inspiratory pressure rise time and inspiratory duration during OLV.

Atelectasis is a common problem during mechanical ventilation. Atelectasis may be caused by a variety of factors, including insufficient inspiratory pressure, short inspiratory time, and insufficient tidal volume [19, 20]. A study on sheep revealed that a brief inspiratory pressure rise time (IRT) led to lung damage [21]. When using the Ingmar ASL5000 in a study to create a passive two-compartment lung model, increasing the IRT helps to balance inspiratory pressure, chamber-specific tidal volume, and volume equilibrium [22]. A study involving 12 infants weighing over 2 kg indicated that varying IRTs during synchronized intermittent positive pressure ventilation or PCV-VG impacted specific ventilation parameters but did not significantly affect oxygenation and carbon dioxide output [23]. Extending the duration of time spent inhaling helped sustain the rise in alveolar pressure, decrease the dead space, enhance arterial oxygen levels and respiratory performance, optimize gas exchange and arterial oxygen levels, and lower the airway pressure [24]. The iso-ratio ventilation group exhibited a lower airway pressure and Pplat, and a higher Cdyn compared to the conventional ventilation group with an inspiration: expiration ratio of 1:2 [25]. In this work, IRT was modified to alter the airflow pattern at the start of inspiration to prevent airway damage from turbulence; in addition to the inspiration: expiration ratio, the end-inspiratory flow was set to “0” to ensure even airflow distribution in the alveoli, which helped decrease atelectasis due to inadequate ventilation and prevented alveolar hyperinflation from hyperventilation. In the current study a reduction in atelectasis occurred, which agrees with data in previous reports [21–25]. These findings suggest that the risk of atelectasis can be effectively reduced by optimizing ventilation strategies. However, pulmonary atelectasis decreased but did not differ between groups. This finding may be due to factors, such as experimental design, sample size, or the range of ventilation strategy adjustments. Future studies could explore this issue further by more rigorous experimental design, larger sample size, or more fine-tuned ventilation parameters.

In a study applying OLV among 1224 adults undergoing lung resection surgery, the PPCs were not influenced by the choice of ventilation mode [26]. According to previous reports, increasing the IRT and extending the duration spent inhaling reduces atelectasis [22–24]. The IRT was changed in the experimental group in the current study and the end-inspiratory flow was set to “0,” which dramatically reduced PPCs in the experimental group compared to the control group and validated that changes in the inspiratory flow rate in the PCV-VG mode may help reduce PPCs [22–24].

Moreover, the respiratory mechanics-related parameters improved, which is consistent with previous reports [24, 27]. As reported by Chong et al., the respiratory parameters did not improve significantly although some of the ventilation parameters did improve significantly [23]. Mechanical ventilation increased the expression of NLRP3 mRNA in alveolar macrophages and caused lung inflammation dependent on NLRP3 inflammatory bodies [28, 29]. High inspiratory airflow during mechanical ventilation causes shear stress along airway and alveolar surfaces, leading to deformation of lung tissue and bronchial cells and the release of substances that promote scarring and inflammation [30, 31]. The current study showed that using the ventilation mode with prolonged inspiratory time and an optimized inspiratory flow rate led to a significant decrease in inflammatory cells, such as the WBC count, in the blood of post-surgery patients. Additionally, levels of the inflammatory mediator, CRP, significantly decreased. These results suggest that this ventilation mode reduces the lung stress response, minimizes lung injury, and provides lung protection.

A significant increase in Cdyn was also a positive observation. Cdyn reflects the elasticity of the lungs during respiration, an improvement of which may indicate that the lungs are more compliant during ventilation (i.e., expand and contract more easily) [32]. Improved lung compliance improves gas exchange, reduces atelectasis, and improves breathing performance. The improved lung compliance at T1, T2, and T4 implies fewer PPCs. Furthermore, the PaO_2_/FiO_2_ did not improve significantly in the experimental group but was significantly higher at T3 while the total complication rate was significantly reduced, which is consistent with previous reports [5, 13, 33]. The optimizing ventilation strategies during OLV were closely associated with fewer PPCs and better perioperative oxygenation [33]. Improvement in the PaO_2_/FiO_2_ may reflect improvement in pulmonary oxygenation function. A decrease in complications may be the result of many factors, including optimization of ventilation parameters, improvement in pulmonary function, and improvement in the patient’s overall condition. Thus, by adjusting the PCV-VG ventilation model parameters, we can effectively reduce the incidence of atelectasis, improve Cdyn, improve oxygenation, and reduce the overall complication rate.

### Limitations

This was a single-center clinical study with a limited number of patients. In addition, the effects of flow rate optimized ventilation mode on lungs at the molecular level should be further elucidated. Changes in the genes and protein levels of inflammatory factors should be determined to better understand the protective mechanism. Moreover, there was a statistical difference in fluid balance between groups but other baseline clinical indicators were not significantly different. Indeed, the above are limitations of this study and may require a larger sample size to eliminate confounding factors.

## Conclusion

Optimizing inspiratory flow rate, using the PCV-VG mode, and adjusting inspiratory pressure rise time and inspiratory time can promote alveolar gas exchange, reduce PPCs, and improve Cdyn in patients undergoing pneumonectomy with OLV.

## Data Availability

The datasets generated during and/or analyzed during the current study are available from the corresponding author on reasonable request.
